# Simultaneous Determination of Caffeine and Paracetamol in Commercial Formulations Using Greener Normal-Phase and Reversed-Phase HPTLC Methods: A Contrast of Validation Parameters

**DOI:** 10.3390/molecules27020405

**Published:** 2022-01-09

**Authors:** Prawez Alam, Faiyaz Shakeel, Abuzer Ali, Mohammed H. Alqarni, Ahmed I. Foudah, Tariq M. Aljarba, Faisal K. Alkholifi, Sultan Alshehri, Mohammed M. Ghoneim, Amena Ali

**Affiliations:** 1 Department of Pharmacognosy, College of Pharmacy, Prince Sattam Bin Abdulaziz University, P.O. Box 173, Al-Kharj 11942, Saudi Arabia; m.alqarni@psau.edu.sa (M.H.A.); a.foudah@psau.edu.sa (A.I.F.); t.aljarba@psau.edu.sa (T.M.A.); 2 Department of Pharmaceutics, College of Pharmacy, King Saud University, P.O. Box 2457, Riyadh 11451, Saudi Arabia; faiyazs@fastmail.fm (F.S.); salshehri1@ksu.edu.sa (S.A.); 3 Department of Pharmacognosy, College of Pharmacy, Taif University, P.O. Box 11099, Taif 21944, Saudi Arabia; abuali@tu.edu.sa; 4 Department of Pharmacology, College of Pharmacy, Prince Sattam Bin Abdulaziz University, P.O. Box 173, Al-Kharj 11942, Saudi Arabia; f.alkholifi@psau.edu.sa; 5 Department of Pharmacy Practice, College of Pharmacy, AlMaarefa University, P.O. Box 71666, Ad Diriyah 13713, Saudi Arabia; mghoneim@mcst.edu.sa; 6 Department of Pharmaceutical Chemistry, College of Pharmacy, Taif University, P.O. Box 11099, Taif 21944, Saudi Arabia; amrathore@tu.edu.sa

**Keywords:** caffeine, greener HPTLC, paracetamol, simultaneous determination, validation

## Abstract

There has been no assessment of the greenness of the described analytical techniques for the simultaneous determination (SMD) of caffeine and paracetamol. As a result, in comparison to the greener normal-phase high-performance thin-layer chromatography (HPTLC) technique, this research was conducted to develop a rapid, sensitive, and greener reversed-phase HPTLC approach for the SMD of caffeine and paracetamol in commercial formulations. The greenness of both techniques was calculated using the AGREE method. For the SMD of caffeine and paracetamol, the greener normal-phase and reversed-phase HPTLC methods were linear in the 50–500 ng/band and 25–800 ng/band ranges, respectively. For the SMD of caffeine and paracetamol, the greener reversed-phase HPTLC approach was more sensitive, accurate, precise, and robust than the greener normal-phase HPTLC technique. For the SMD of caffeine paracetamol in commercial PANEXT and SAFEXT tablets, the greener reversed-phase HPTLC technique was superior to the greener normal-phase HPTLC approach. The AGREE scores for the greener normal-phase and reversed-phase HPTLC approaches were estimated as 0.81 and 0.83, respectively, indicated excellent greenness profiles for both analytical approaches. The greener reversed-phase HPTLC approach is judged superior to the greener normal-phase HPTLC approach based on numerous validation parameters and pharmaceutical assays.

## 1. Introduction

**Figure 1:**
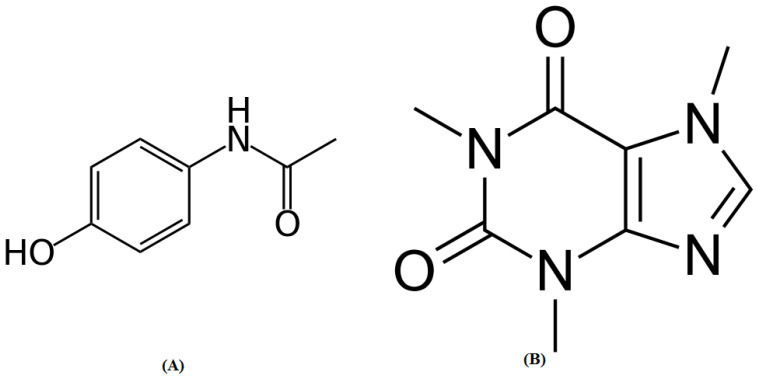
Chemical structures of (**A**) paracetamol and (**B**) caffeine.

Paracetamol (Figure 1A) is the commonly administered anti-inflammatory and antipyretic medicine, especially in case of pediatric and geriatric patients [1,2]. It is commercially available in a wide range of dosage forms [2]. Caffeine (Figure 1B) is a pseudo-alkaloidal drug that is commonly used in combination with paracetamol [3,4]. The combination of paracetamol and caffeine is the world’s most widely used combination [4]. As a result, the qualitative and quantitative standardization of caffeine and paracetamol in commercially available formulations is necessary. 

An extensive literature search revealed various analytical approaches for the simultaneous determination (SMD) of caffeine and paracetamol in commercial formulations and biological fluids. For the SMD of caffeine and paracetamol in commercial formulations, different spectrometry techniques involving various chemical procedures, such as derivatization have been used [5,6,7,8,9,10]. For the SMD of caffeine and paracetamol in various commercial dosage forms, several high-performance liquid chromatography (HPLC) techniques have been used [4,11,12,13,14,15,16,17,18,19]. Caffeine and paracetamol were also quantified simultaneously in a human plasma sample using a HPLC method [19]. For the SMD of caffeine and paracetamol in human plasma samples, a liquid-chromatography mass-spectrometry (LC–MS) technique was also used [20]. For the SMD of caffeine and paracetamol in their pure forms and formulations, certain high-performance thin-layer chromatography (HPTLC) techniques have been used [21,22,23]. Various voltammetry-based approaches have also been applied for the SMD of caffeine and paracetamol in their dosage forms [24,25,26,27]. Dual-mode gradient HPLC and HPTLC methods have also been used for the SMD of caffeine and paracetamol in the presence of paracetamol impurities [28]. The electrospray laser desorption ionization mass spectrometry technique was also utilized for the SMD of caffeine and paracetamol in tablets [29]. An electrochemical cell-on-a-chip device fabricated using 3D-printing technology was also used for the SMD of caffeine and paracetamol [30]. A genetic algorithm based on wavelength selection was also applied for the SMD of caffeine and paracetamol [31]. Some other approaches, such as near-infrared spectrometry [32], flow-injection spectrometry [33], micellar liquid chromatography [34], and micellar electrokinetic capillary chromatography [35] approaches were also proposed for the SMD of caffeine and paracetamol in their dosage forms. Published reports on the SMD of caffeine and paracetamol suggested various analytical approaches for their analysis. However, the greenness scale of any of the reported analytical approach was not estimated. In addition, greener HPTLC approaches have not been utilized for the SMD of caffeine and paracetamol. For the estimation of the greenness scale, different quantitative analytical methodologies have been presented [36,37,38,39,40]. For the estimation of the greenness scale, only the “Analytical Greenness (AGREE)” analytical approach considers all twelve green analytical chemistry (GAC) principles [38]. As a result, the AGREE analytical methodology was applied for the estimation of greenness scale of the greener normal-phase and reversed-phase HPTLC approaches [38]. 

In comparison to the greener normal-phase HPTLC approach, the current study intends to establish and validate a rapid, sensitive, and greener reversed-phase HPTLC approach for the SMD of caffeine and paracetamol in commercial formulations. Following “The International Council for Harmonization (ICH)” Q2-R1 recommendations, the greener normal-phase and reversed-phase HPTLC methods for the SMD of caffeine and paracetamol were validated [41].

## 2. Results and Discussion

### 2.1. Method Development

For the development of a suitable band for the SMD of caffeine and paracetamol using the greener normal-phase HPTLC technique, various amounts of ethyl acetate (EA) and ethanol (E), including EA/E (50:50, *v*/*v*), EA/E (60:40, *v*/*v*), EA/E (70:30, *v*/*v*), EA/E (80:20, *v*/*v*), EA/E (85:15, *v*/*v*), and EA/E (90:10, *v*/*v*) were studied as the greener mobile phases. 

**Figure 2:**
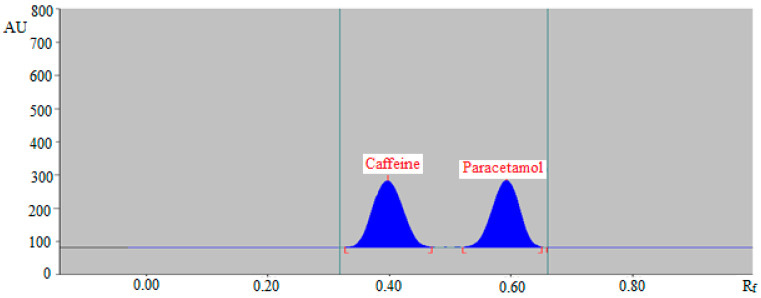
Normal-phase high-performance thin-layer chromatography (HPTLC) chromatogram of standard caffeine and paracetamol.

The greener mobile phases, such as EA/E (50:50, *v*/*v*), EA/E (60:40, *v*/*v*), EA/E (70:30, *v*/*v*), EA/E (80:20, *v*/*v*), and EA/A (90:10, *v*/*v*) revealed poor chromatographic peaks of caffeine and paracetamol with high asymmetry factor (As) for caffeine (As > 1.25) and paracetamol (As > 1.30). When the greener mobile phase EA/E (85:15, *v*/*v*) was evaluated, it was discovered that this greener mobile phase provided well-resolved and intact chromatographic peaks for caffeine at a retardation factor of (R_f_) = 0.40 ± 0.01 and for paracetamol of R_f_ = 0.59 ± 0.02 (Figure 2). Caffeine and paracetamol were also predicted to have As values of 1.06 and 1.08, respectively, which are very trustworthy. As a consequence, the EA/E (85:15, *v*/*v*) was chosen as the final mobile phase for the SMD of caffeine and paracetamol in commercial tablets utilizing the greener normal-phase HPTLC method.

**Figure 3:**
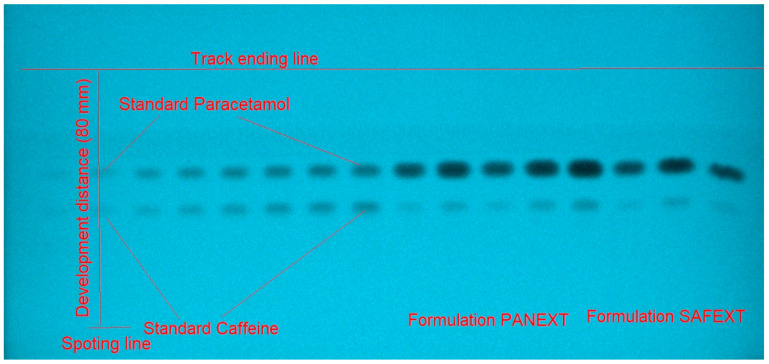
Developed thin-layer chromatography (TLC) plate for standard caffeine, standard paracetamol, commercial tablets PANEXT, and commercial tablets SAFEXT developed using ethanol (E)/water (W) (50:50 *v*/*v*) as the greener mobile phase for the greener reversed-phase HPTLC method.

For the development of a suitable band for the SMD of caffeine and paracetamol using the greener reversed-phase HPTLC technique, various amounts of E and water (W), including E/W (50:50, *v*/*v*), E/W (60:40, *v*/*v*), E/W (70:30, *v*/*v*), E/W (80:20, *v*/*v*), and E/W (90:10, *v*/*v*) were studied as the greener mobile phases. All of the green mobile phases investigated were created under chamber saturation conditions (Figure 3). 

**Figure 4:**
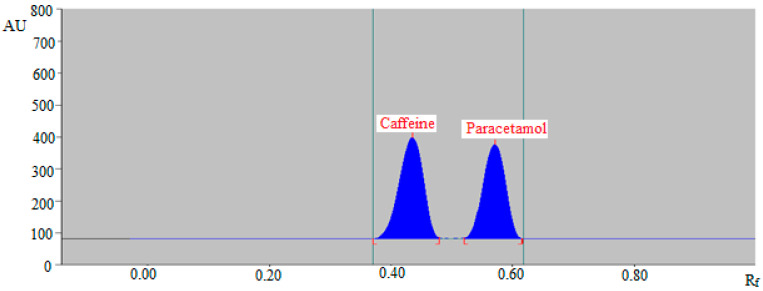
Reversed-phase HPTLC chromatogram of standard caffeine and paracetamol.

The greener mobile phases, such as E/W (60:40, *v*/*v*), E/W (70:30, *v*/*v*), E/W (80:20, *v*/*v*), and E/W (90:10, *v*/*v*) revealed poor chromatographic peaks of caffeine and paracetamol with poor As for caffeine (As > 1.30) and paracetamol (As > 1.35). When the greener mobile phase E/W (50:50, *v*/*v*) was evaluated, it was discovered that this greener mobile phase provided well-resolved and intact chromatographic peaks of caffeine at R_f_ = 0.43 ± 0.01 and of paracetamol at R_f_ = 0.57 ± 0.02 (Figure 4). Caffeine and paracetamol were also predicted to have As values of 1.10 and 1.09, respectively, which are very trustworthy. As a consequence, the E/W (50:50, *v*/*v*) was chosen as the final mobile phase for the SMD of caffeine and paracetamol in commercial tablets utilizing the greener reversed-phase HPTLC method. The maximum response was obtained at a wavelength of 260 nm for caffeine and paracetamol when the spectral bands for caffeine and paracetamol were recorded using densitometry mode. As a result, the whole SMD of caffeine and paracetamol took place at 260 nm.

### 2.2. Method Validation

The ICH-Q2-R1 guidelines were used to estimate various parameters for the SMD of caffeine and paracetamol [41]. The results of the linear regression analysis of caffeine and paracetamol calibration curves utilizing the greener normal-phase HPTLC technique are summarized in Table 1. Caffeine and paracetamol calibration curves were linear in the 50–500 ng/band range for both drugs. Caffeine and paracetamol’s determination coefficients (R^2^) were found to be 0.9928 and 0.9970, respectively. Caffeine and paracetamol’s regression coefficients (R) were found to be 0.9963 and 0.9984, respectively. The values of R^2^ and R were highly significant for both the compounds (*p* < 0.05). These findings suggested a strong link between the concentration and measured response of caffeine and paracetamol. All these findings indicated the reliability of the greener normal-phase HPTLC approach for the SMD of caffeine and paracetamol. 

The resulting data for the linear regression analysis of caffeine and paracetamol calibration curves utilizing the greener reversed-phase HPTLC technique are summarized in Table 2. Caffeine and paracetamol calibration curves were linear in the 25–800 ng/band range for both drugs. Caffeine and paracetamol’s R^2^ were found to be 0.9976 and 0.9966, respectively. Caffeine and paracetamol’s R were found to be 0.9987 and 0.9982, respectively. The values of R^2^ and R were highly significant for both the compounds (*p* < 0.05). These findings again suggested a strong link between the concentration and measured response of caffeine and paracetamol. All these findings indicated the reliability of the greener reversed-phase HPTLC technique for the SMD of caffeine and paracetamol. However, the greener reversed-phase HPTLC technique was more linear than the greener normal-phase HPTLC technique. 

The parameters of the system appropriateness for the greener normal-phase HPTLC methodology are summarized in Table 3. For the SMD of caffeine and paracetamol, the R_f_, As, and number of theoretical plates per meter (N/m) for the greener normal-phase HPTLC technique were determined to be satisfactory. The parameters of the system appropriateness for the greener reversed-phase HPTLC methodology are summarized in Table 4. For the SMD of caffeine and paracetamol, the R_f_, As, and N/m for the greener reversed-phase HPTLC technique were also determined to be satisfactory.

For assessing caffeine and paracetamol, the percent of recovery was utilized to estimate the accuracy of the greener normal-phase and reversed-phase HPTLC techniques. The accuracy evaluation results for the greener normal-phase HPTLC technique are summarized in Table 5. Using the greener normal-phase HPTLC technique, the percent recoveries of caffeine and paracetamol at three separate quality control (QC) samples were expected to be 97.13–104.88 and 96.57–103.23 percent, respectively. The accuracy evaluation results for the greener reversed-phase HPTLC technique are summarized in Table 6. Using the greener reversed-phase HPTLC technique, the percent recoveries of caffeine and paracetamol at three separate QC samples were expected to be 98.84–100.62 and 98.60–101.50 percent, respectively. These results showed that both analytical techniques were accurate for the SMD of caffeine and paracetamol. For the SMD of caffeine and paracetamol, however, the greener reversed-phase HPTLC methodology was more accurate than the greener normal-phase HPTLC methodology. 

The precision of the greener normal-phase and reversed-phase HPTLC techniques was investigated as intra/inter-assay precision and given as a percent of the coefficient of variation (CV) for the SMD of caffeine and paracetamol. Table 7 summarizes the results of intra/inter-day precisions for the SMD of caffeine and paracetamol using the greener normal-phase HPTLC technique. The percent CVs of caffeine and paracetamol for the intra-day variation were estimated as 1.30–2.39 and 1.91–3.42 percent, respectively. The percent CVs of caffeine and paracetamol for inter-day variation were estimated as 1.51–2.55 and 1.86–3.56 percent, respectively. Table 8 summarizes the results of intra/inter-day precisions for the SMD of caffeine and paracetamol using the greener reversed-phase HPTLC technique. The percent CVs of caffeine and paracetamol for the intra-day variation were estimated as 0.40–0.85 and 0.52–0.96 percent, respectively. The percent CVs of caffeine and paracetamol for inter-day variation were estimated as 0.42–0.78 and 0.55–1.03 percent, respectively. These findings indicated that both the analytical approaches were precise for the SMD of caffeine and paracetamol. However, the greener reversed-phase HPTLC methodology was more precise than the greener normal-phase HPTLC methodology for the SMD of caffeine and paracetamol. 

By introducing slight deliberate modifications in the greener mobile phase components, the durability of the greener normal-phase and reversed-phase HPTLC techniques for the SMD of caffeine and paracetamol was examined. Table 9 summarizes the results of robustness evaluation using the greener normal-phase HPTLC approach. The percent CVs for caffeine and paracetamol were estimated as 2.17–3.33 and 2.48–2.64 percent, respectively. Caffeine and paracetamol R_f_ values were also estimated to be 0.39–0.41 and 0.58–0.60, respectively.

Table 10 summarizes the results of robustness evaluation utilizing the greener reversed-phase HPTLC methodology. The percent CVs for caffeine and paracetamol were estimated as 0.91–0.94 and 0.95–1.04 percent, respectively. Caffeine and paracetamol R_f_ values were also estimated to be 0.42–0.44 and 0.56–0.58, respectively. These results showed that both analytical techniques were reliable for the SMD of caffeine and paracetamol. For the SMD of caffeine and paracetamol, however, the greener reversed-phase HPTLC approach was more robust than the greener normal-phase HPTLC approach. 

The “limit of detection (LOD) and limit of quantification (LOQ)” were used to evaluate the sensitivity of the greener normal-phase and reversed-phase HPTLC methods for the SMD of caffeine and paracetamol. The predicted values of “LOD and LOQ” for caffeine and paracetamol utilizing the greener normal-phase HPTLC technique are summarized in Table 1. Using the greener normal-phase HPTLC technique, the “LOD and LOQ” for caffeine were estimated to be 16.84 *±* 0.27 and 50.52 *±* 0.81 ng/band, respectively. Using the greener normal-phase HPTLC technique, the “LOD and LOQ” for paracetamol were estimated to be 17.05 ± 0.31 and 51.15 ± 0.93 ng/band, respectively. The predicted values of “LOD and LOQ” for caffeine and paracetamol utilizing the greener reversed-phase HPTLC technique are summarized in Table 2. Utilizing the reversed-phase HPTLC technique, the “LOD and LOQ” for caffeine were estimated to be 8.52 *±* 0.12 and 25.56 *±* 0.36 ng/band, respectively. Using the greener reversed-phase HPTLC technique, the “LOD and LOQ” for paracetamol were estimated to be 8.71 ± 0.13 and 26.13 ± 0.39 ng/band, respectively. These data suggested that both analytical techniques were sensitive enough for the SMD of caffeine and paracetamol. For the SMD of caffeine and paracetamol, however, the reversed-phase HPTLC methodology was more sensitive than the normal-phase HPTLC methodology.

**Figure 5:**
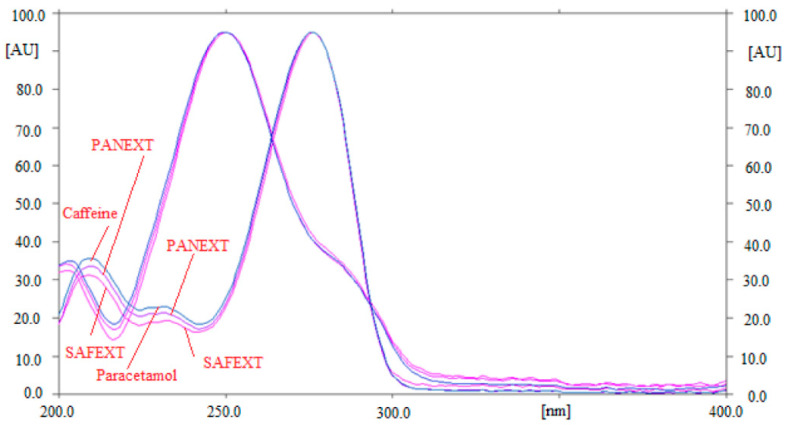
Superimposed ultra-violet (UV) absorption spectra of standard caffeine and paracetamol and caffeine and paracetamol in PANEXT and SAFEXT.

By comparing the R_f_ values and superimposed ultra-violet (UV)-absorption spectra of caffeine and paracetamol in the commercial tablets PANEXT and SAFEXT with that of standards caffeine and paracetamol, the specificity of the greener HPTLC approach for the SMD of caffeine and paracetamol was assessed. The overlaid UV spectra of standards caffeine and paracetamol, as well as caffeine and paracetamol in the commercial tablets PANEXT and SAFEXT, are shown in Figure 5.

At a wavelength of 260 nm, the maximum densitometric responses of caffeine and paracetamol in standards and the commercial tablets PANEXT and SAFEXT were recorded. The specificity of the greener HPTLC technique for the SMD of caffeine and paracetamol was demonstrated by the identical UV spectra, R_f_ data, and wavelengths of caffeine and paracetamol in standards and the commercial tablets PANEXT and SAFEXT.

### 2.3. Application of Greener Normal-Phase and Reversed-Phase HPTLC Aapraches in the SMD of Caffeine and Paracetamol in Commercial Tablets 

**Figure 6:**
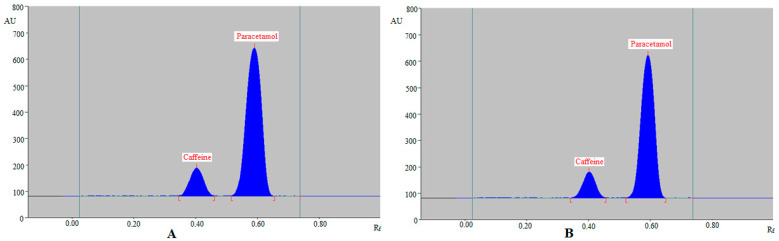
Normal-phase HPTLC chromatograms of caffeine and paracetamol in (**A**) commercial tablets PANEXT and (**B**) commercial tablets SAFEXT.

For the SMD of caffeine and paracetamol in their commercial formulation, the greener normal-phase and reversed-phase HPTLC techniques were used as an alternative to regular analytical approaches. The chromatograms of caffeine and paracetamol from commercial tablets were identified by comparing the TLC spots at R_f_ = 0.40 ± 0.01 for caffeine and R_f_ = 0.59 ± 0.02 for paracetamol in comparison with those of standards for caffeine and paracetamol using the greener normal-phase HPTLC approach. Figure 6 summarizes the recorded chromatograms of caffeine and paracetamol in the commercial tablets PANEXT (Figure 6A) and SAFEXT (Figure 6B), which showed identical peaks of caffeine and paracetamol to those of standards for caffeine and paracetamol in both the commercial tablets. 

**Figure 7:**
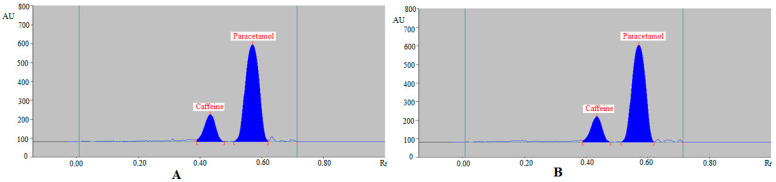
Reversed-phase HPTLC chromatograms of caffeine and paracetamol in (**A**) commercial tablets PANEXT and (**B**) commercial tablets SAFEXT.

The chromatograms of caffeine and paracetamol from commercial tablets were identified by comparing their TLC spots at R_f_ = 0.43 ± 0.01 for caffeine and R_f_ = 0.57 ± 0.02 for paracetamol with those of standards caffeine and paracetamol using the greener reversed-phase HPTLC approach. Figure 7 summarizes the recorded chromatograms of caffeine and paracetamol in the commercial tablets PANEXT (Figure 7A) and SAFEXT (Figure 7B), which also showed identical peaks of caffeine and paracetamol to those of standards caffeine and paracetamol in both commercial tablets. 

Using the greener normal-phase HPTLC technique, the percent assays of caffeine in the commercial PANEXT and SAFEXT tablets were estimated to be 91.23 ± 1.14 and 92.45 ± 1.22 percent, respectively. Using the greener normal-phase HPTLC technique, the percent assays of paracetamol in commercial PANEXT and SAFEXT tablets were estimated to be 89.41 ± 1.04 and 91.13 ± 1.06 percent, respectively. Using the greener reversed-phase HPTLC technique, the percent assays of caffeine in commercial PANEXT and SAFEXT tablets were estimated to be 98.51 ± 1.42 and 101.12 ± 1.53 percent, respectively. Using the greener reversed-phase HPTLC technique, the percent assays of paracetamol in commercial PANEXT and SAFEXT tablets were estimated to be 99.42 ± 1.45 and 100.64 ± 1.49 percent, respectively. The greener normal-phase and reversed-phase HPTLC methods were shown to be suitable for the SMD of caffeine and paracetamol in commercial formulations. However, for the SMD of caffeine and paracetamol in commercial formulations, the reversed-phase HPTLC methodology was more reliable than the normal-phase HPTLC methodology.

### 2.4. Greenness Estimation Using AGREE 

**Figure 8:**
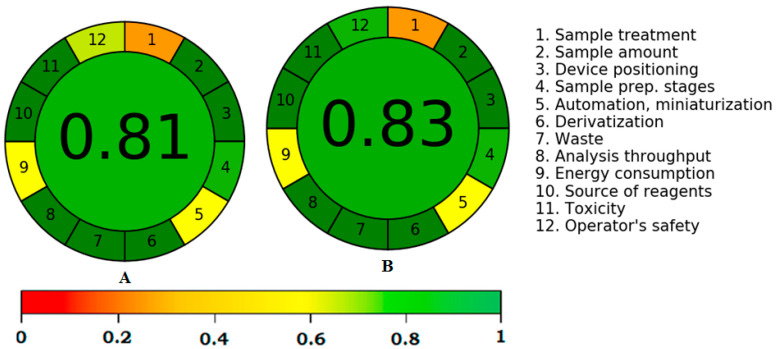
“Analytical GREEnness (AGREE)” scale for (**A**) greener normal-phase HPTLC and (**B**) greener reversed-phase HPTLC methods.

Various quantitative approaches are available for the greenness estimation of analytical approaches [36,37,38,39,40]. However, only AGREE applies all twelve GAC principles for greenness estimation [38]. As a result, the greenness of the greener normal-phase and reversed-phase HPTLC approaches was estimated by “AGREE: The Analytical Greenness Calculator (version 0.5, Gdansk University of Technology, Gdansk, Poland, 2020)”. The typical diagram for the AGREE scale of the greener normal-phase and reversed-phase HPTLC techniques is shown in Figure 8. The AGREE scale was estimated to be 0.81 and 0.83 for the greener normal-phase and reversed-phase HPTLC methods, respectively. These findings indicated the excellent greenness nature of the greener normal-phase and reversed-phase HPTLC approaches for the SMD of caffeine and paracetamol in their commercial formulations.

## 3. Materials and Methods

### 3.1. Materials

The standards of caffeine and paracetamol were provided by “Sigma Aldrich (St. Louis, MO, USA)”. HPLC-grades E and EA were provided by “E-Merck (Darmstadt, Germany)”. The W was obtained from the Milli-Q unit in the laboratory. The commercial tablets PANEXT and SAFEXT were obtained from the pharmacy shop in “Al-Kharj, Saudi Arabia”. All other solvents utilized were of analytical grades.

### 3.2. Instrumentation and Analytical Procedures 

The “HPTLC CAMAG TLC system (CAMAG, Muttenz, Switzerland)” was used for the SMD of caffeine and paracetamol in their standards and commercial tablets. The sample solutions were spotted as 6-mm bands utilizing a “CAMAG Automatic TLC Sampler 4 (ATS4) Sample Applicator (CAMAG, Geneva, Switzerland)”. The “CAMAG microliter Syringe (Hamilton, Bonaduz, Switzerland)” was linked with sample applicator. The application rate for the SMD of caffeine and paracetamol was fixed at 150 nL/s. Under linear ascending mode, the TLC plates were developed in a “CAMAG automated developing chamber 2 (ADC2) (CAMAG, Muttenz, Switzerland)” at a distance of 80 mm. For 30 min at 22 °C, the development chamber was saturated with vapors of greener mobile phases. Caffeine and paracetamol were detected using a wavelength of 260 nm. The slit size (band length × width) and scanning rate were both set at 4 mm × 0.45 mm and 20 mm/s, respectively. Three or six replicates were used for each estimation. The software used was “WinCAT’s (version 1.4.3.6336, CAMAG, Muttenz, Switzerland)”. 

The greener normal-phase and reversed-phase HPTLC methodologies used the same instrumentation and analytical procedures as the normal-phase and reversed-phase HPTLC approaches. The TLC plates and the greener mobile phase components were found to be the most significant differences between the two procedures. In the greener normal-phase HPTLC technique, the TLC plates were glass plates (plate size: 10 cm × 20 cm) pre-coated with normal-phase silica gel (particle size: 5 µm) 60F254S plates, but in the greener reversed-phase HPTLC approach, the TLC plates were glass plates (plate size: 10 cm × 20 cm) pre-coated with reversed-phase silica gel (particle size: 5 µm) 60F254S plates. In both cases, the polymer-binder plate was not used. In the greener normal-phase HPTLC approach, the greener mobile phase was EA/E (85:15, *v*/*v*); however, in the greener reversed-phase HPTLC approach, the greener mobile phase was E/W (50:50, *v*/*v*).

### 3.3. Calibration Curves and QC Sample for Caffeine and Paracetamol

Caffeine and paracetamol stock solutions were made individually by dispensing the requisite amounts of both molecules in the specified amount of respective mobile phase, resulting in a final stock solution of 100 µg/mL for both compounds. The concentrations in the 50–500 ng/band range for caffeine and paracetamol were generated using the greener normal-phase HPTLC methodology and the 25–800 ng/band range for caffeine and paracetamol using the greener reversed-phase HPTLC methodology by diluting variable volumes of caffeine or paracetamol stock solution with the respective mobile phase. For the normal-phase HPTLC methodology, 200 µL of each concentration of caffeine and paracetamol were put to normal-phase TLC plates and reversed-phase TLC plates for the reversed-phase HPTLC methodology. Using both analytical techniques, the spot area of each concentration of caffeine and paracetamol was measured. Caffeine and paracetamol calibration curves were created by graphing the concentrations of both drugs against the observed spot area in six repeats (*n* = 6). For the determination of various validation parameters, three distinct QC samples were prepared freshly.

### 3.4. Processing of Samples for the SMD of Caffeine and Paracetamol in Commercial Tablets 

Ten commercial tablets (each containing 65 mg of caffeine and 500 mg of paracetamol) were weighed and the average weights were computed for the SMD of caffeine and paracetamol in PANEXT and SAFEXT. Each brand’s tablets were coarsely crushed and powdered. A portion of each brand’s powder was dissolved in 100 mL of the relevant mobile phase. For the greener normal-phase and reversed-phase HPTLC methods, 1 mL of this solution of each brand of tablet was diluted again using 10 mL of the corresponding mobile phase. The prepared solutions of PANEXT and SAFEXT commercial tablets were filtered and sonicated for around ten minutes to remove any undissolved excipients. Using the greener normal-phase and reversed-phase HPTLC methods, the generated solutions were used to determine caffeine and paracetamol in commercial tablets PANEXT and SAFEXT.

### 3.5. Analytical Method Validation

Utilizing the ICH-Q2-R1 recommendations, the normal-phase and reversed-phase HPTLC techniques for the SMD of caffeine and paracetamol were validated for various parameters [41]. By graphing the concentrations of caffeine and paracetamol against their measured spot area, the linearity range for caffeine and paracetamol was discovered. The normal-phase HPTLC approach’s linearity for caffeine and paracetamol was evaluated in the 50–500 ng/band range (*n* = 6). For the reversed-phase HPTLC method, the linearity for caffeine and paracetamol was evaluated in the 25–800 ng/band range (*n* = 6). 

The calculation of R_f_, As, and N/m was used to evaluate the parameters for the system acceptability for the greener normal-phase and reversed-phase HPTLC techniques for the SMD of caffeine and paracetamol. For both analytical approaches, the R_f_, As, and N/m data were computed utilizing their reported equations [39].

The percent recovery was utilized to examine the accuracy of the normal-phase and reversed-phase HPTLC methods for the SMD of caffeine and paracetamol. For caffeine and paracetamol, the accuracy of the greener normal-phase HPTLC technique was tested at three QC levels: lower QC (LQC; 100 ng/band), middle QC (MQC; 300 ng/band), and high QC (HQC; 500 ng/band). For caffeine and paracetamol, the accuracy of the greener reversed-phase HPTLC technique was tested at three QC levels: LQC (50 ng/band), MQC (300 ng/band), and HQC (800 ng/band). Using both analytical techniques, the percent of recovery for caffeine and paracetamol (*n* = 6) was assessed at each QC level. 

Intra/inter-assay precision was measured for the greener normal-phase and reversed-phase HPTLC methods for caffeine and paracetamol. Quantitation of newly prepared caffeine and paracetamol solutions at LQC, MQC, and HQC on the same day for both analytical techniques (*n* = 6), was used to examine intra-assay variation for caffeine and paracetamol. Quantitation of freshly prepared solutions at LQC, MQC, and HQC on three consecutive days for both analytical techniques (*n* = 6) was used to investigate inter-assay variation for caffeine and paracetamol.

For both analytical techniques, the robustness for caffeine and paracetamol was evaluated by making some slight purposeful modification in the mobile phase composition. The greener mobile phase EA/E (85:15, *v*/*v*) for caffeine and paracetamol was altered to EA/E (87:13, *v*/*v*) and EA/E (83:17, *v*/*v*) for the greener normal-phase HPTLC technique, and the variations in chromatographic response and R_f_ values were recorded (*n* = 6). The greener mobile phase E/W (50:50, *v*/*v*) for caffeine and paracetamol was altered to E/W (52:48, *v*/*v*) and E/W (48:52, *v*/*v*) for the greener reversed-phase HPTLC technique, and the variations in chromatographic response and R_f_ values were recorded (*n* = 6).

By using a “standard deviation” technique, the sensitivity of the greener normal-phase and reversed-phase HPTLC approaches for caffeine and paracetamol was examined as “LOD and LOQ”. Caffeine and paracetamol “LOD and LOQ” were computed using their published equations for both analytical procedures (*n* = 6) [41].

The R_f_ values and UV spectra of caffeine and paracetamol in commercial tablets PANEXT and SAFEXT were compared with those of standards caffeine and paracetamol to determine the specificity of the greener normal-phase and reversed-phase HPTLC methods for caffeine and paracetamol.

### 3.6. Application of Greener Normal-Phase and Reversed-Phase HPTLC Approaches in the SMD of Caffeine and Paracetamol in Commercial Tablets

For the normal-phase HPTLC technique, the obtained solutions of the commercial tablets PANEXT and SAFEXT were put on normal-phase TLC plates and on reversed-phase TLC plates for the reversed-phase HPTLC technique. For all analytical techniques, the chromatographic responses were documented using the identical experimental circumstances employed for the SMD of standards caffeine and paracetamol (*n* = 3). For both analytical procedures, the quantities of caffeine and paracetamol in commercial tablets were approximated using the calibration curves for caffeine and paracetamol.

### 3.7. Greenness Estimation Using AGREE 

The AGREE technique [38] was utilized to assess the greenness scale for the normal-phase and reversed-phase HPTLC procedures for the SMD of caffeine and paracetamol. The AGREE scales (0.0–1.0) for the greener normal-phase and reversed-phase HPTLC approaches was estimated utilizing “AGREE: The Analytical Greenness Calculator (version 0.5, Gdansk University of Technology, Gdansk, Poland, 2020)” for both the analytical approaches. 

## 4. Conclusions

The literature lacks greener analytical techniques for the SMD of caffeine and paracetamol. As a result, compared to the greener normal-phase HPTLC approach, this research was carried out to develop and validate the rapid, sensitive, and greener reversed-phase HPTLC approach for the SMD of caffeine and paracetamol in their commercial tablets. For the SMD of caffeine and paracetamol, the greener reversed-phase HPTLC approach is more linear, accurate, precise, robust, and sensitive than the greener normal-phase HPTLC approach. The quantities of caffeine and paracetamol in commercial tablets PANEXT and SAFEXT were found to be significantly higher using the reversed-phase HPTLC methodology compared with the normal-phase HPTLC methodology. The AGREE estimation showed the excellent green properties of both the analytical approaches. For the SMD of caffeine and paracetamol in commercial formulations, the greener reversed-phase HPTLC approach has been presented superior to the greener normal-phase HPTLC approach based on different validation criteria and pharmaceutical assays.

## Figures and Tables

**Table 1:** Results of the linear regression analysis for the simultaneous determination (SMD) of caffeine and paracetamol using the greener normal-phase high-performance thin-layer chromatography (HPTLC) method (mean ± SD; *n* = 6).

Parameters	Caffeine	Paracetamol
linearity range (ng/band)	50–500	50–500
R^2^	0.9928	0.9970
R	0.9963	0.9984
slope ± SD	19.36 ± 0.96	18.05 ± 0.54
intercept ± SD	387.62 ± 4.38	95.66 ± 2.14
standard error of slope	0.39	0.22
standard error of intercept	1.78	0.87
95% confidence interval of slope	17.68–21.05	17.10–19.00
95% confidence interval of intercept	379.92–395.31	91.90–99.42
LOD ± SD (ng/band)	16.84 ± 0.27	17.05 ± 0.31
LOQ ± SD (ng/band)	50.52 ± 0.81	51.15 ± 0.93

R^2^: determination coefficient; R: regression coefficient; LOD: limit of detection; LOQ: limit of quantification.

**Table 2:** Results for linear regression analysis for the SMD of caffeine and paracetamol using the greener reversed-phase HPTLC method (mean ± SD; *n* = 6).

Parameters	Caffeine	Paracetamol
linearity range (ng/band)	25–800	25–800
R^2^	0.9976	0.9966
R	0.9987	0.9982
slope ± SD	20.54 ± 1.05	17.12 ± 0.47
intercept ± SD	833.46 ± 7.51	696.63 ± 6.21
standard error of slope	0.42	0.19
standard error of intercept	3.06	2.53
95% confidence interval of slope	18.70–22.38	16.29–17.94
95% confidence interval of intercept	820.26–846.65	685.71–707.54
LOD ± SD (ng/band)	8.52 ± 0.12	8.71 ± 0.13
LOQ ± SD (ng/band)	25.56 ± 0.36	26.13 ± 0.39

R^2^: determination coefficient; R: regression coefficient; LOD: limit of detection; LOQ: limit of quantification.

**Table 3:** System suitability parameters in terms of retardation factor (R_f_), asymmetry factor (As), and a number of theoretical plates per meter (N/m) of caffeine and paracetamol for the greener normal-phase HPTLC method (mean ± SD; *n* = 3).

Parameters	Caffeine	Paracetamol
R_f_	0.40 *±* 0.01	0.59 *±* 0.02
As	1.06 *±* 0.02	1.08 *±* 0.03
N/m	5245 *±* 5.81	4978 *±* 5.19

**Table 4:** The R_f_, As, and N/m values of caffeine and paracetamol for the greener reversed-phase HPTLC method (mean ± SD; *n* = 3).

Parameters	Caffeine	Paracetamol
R_f_	0.43 *±* 0.01	0.57 *±* 0.02
As	1.10 *±* 0.03	1.09 *±* 0.02
N/m	5182 *±* 5.92	5367 *±* 6.32

**Table 5:** Measurement of the accuracy of caffeine and paracetamol for the greener normal-phase HPTLC method (mean ± SD; *n* = 6).

Conc. (ng/Band)	Conc. Found (ng/Band) ± SD	Recovery (%)	CV (%)
	Caffeine		
100	97.13 ± 2.24	97.13	2.30
300	314.65 ± 4.87	104.88	1.54
500	492.31 ± 6.13	98.46	1.24
	Paracetamol		
100	103.23 ± 3.12	103.23	3.02
300	289.71 ± 7.12	96.57	2.45
500	514.41 ± 9.12	102.88	1.77

CV: coefficient of variance.

**Table 6:** Measurement of the accuracy of caffeine and paracetamol for the greener reversed-phase HPTLC method (mean ± SD; *n* = 6).

Conc. (ng/Band)	Conc. Found (ng/Band) ± SD	Recovery (%)	CV (%)
	Caffeine		
50	50.31 ± 0.41	100.62	0.81
300	296.54 ± 1.45	98.84	0.48
800	795.61 ± 3.45	99.45	0.43
	Paracetamol		
50	49.18 ± 0.41	98.36	0.83
300	304.51 ± 1.64	101.50	0.53
800	807.54 ± 4.15	100.94	0.51

CV: coefficient of variance.

**Table 7:** Assessment of intra/inter-day precision of caffeine and paracetamol for the greener normal-phase HPTLC method (mean ± SD; *n* = 6).

Conc. (ng/Band)	Intraday Precision			Interday Precision		
	Conc. (ng/Band) ± SD	Standard Error	CV (%)	Conc. (ng/Band) ± SD	Standard Error	CV (%)
Caffeine						
100	96.54 ± 2.31	0.94	2.39	103.65 ± 2.65	1.08	2.55
300	292.97 ± 5.02	2.04	1.71	291.98 ± 5.61	2.29	1.92
500	512.45 ± 6.68	2.72	1.30	483.27 ± 7.31	2.98	1.51
Paracetamol						
100	96.89 ± 3.32	1.35	3.42	95.61 ± 3.41	1.39	3.56
300	313.56 ± 7.52	3.07	2.39	286.51 ± 7.59	3.09	2.64
500	486.67 ± 9.32	3.80	1.91	516.41 ± 9.61	3.92	1.86

CV: coefficient of variance.

**Table 8:** Assessment of intra/inter-day precision of caffeine and paracetamol for the greener reversed-phase HPTLC method (mean ± SD; *n* = 6).

Conc. (ng/Band)	Intraday Precision			Interday Precision		
	Conc. (ng/Band) ± SD	Standard Error	CV (%)	Conc. (ng/Band) ± SD	Standard Error	CV (%)
Caffeine						
50	50.42 ± 0.43	0.17	0.85	49.61 ± 0.39	0.15	0.78
300	303.21 ± 1.51	0.61	0.49	297.54 ± 1.31	0.53	0.44
800	806.31 ± 3.61	1.47	0.40	797.61 ± 3.40	1.38	0.42
Paracetamol						
50	49.54 ± 0.48	0.19	0.96	50.21 ± 0.52	0.21	1.03
300	305.61 ± 1.78	0.72	0.58	296.54 ± 1.81	0.73	0.61
800	794.65 ± 4.21	1.71	0.52	804.61 ± 4.47	1.82	0.55

CV: coefficient of variance.

**Table 9:** Results of robustness analysis of caffeine and paracetamol for the greener normal-phase HPTLC method (mean ± SD; *n* = 6).

Conc. (ng/Band)	Mobile Phase Composition (Ethyl Acetate/Ethanol)			Results		
	Original	Used		(ng/Band) ± SD	% CV	R_f_
Caffeine						
		87:13	+2.0	294.98 ± 6.43	2.17	0.39
300	85:15	85:15	0.0	302.14 ± 6.87	2.27	0.40
		83:17	−2.0	305.61 ± 7.13	2.33	0.41
Paracetamol						
		87:13	+2.0	287.21 ± 7.14	2.48	0.58
300	85:15	85:15	0.0	291.34 ± 7.72	2.64	0.59
		83:17	−2.0	304.51 ± 8.02	2.63	0.60

CV: coefficient of variance; R_f_: retardation factor.

**Table 10:** Results of robustness analysis of caffeine and paracetamol for the greener reversed-phase HPTLC method (mean ± SD; *n* = 6).

Conc. (ng/Band)	Mobile Phase Composition (Ethanol/Water)			Results		
	Original	Used		(ng/Band) ± SD	% CV	R_f_
Caffeine						
		52:48	+2.0	296.31 ± 2.71	0.91	0.42
300	50:50	50:50	0.0	303.54 ± 2.82	0.92	0.43
		48:52	−2.0	306.87 ± 2.91	0.94	0.44
Paracetamol						
		52:48	+2.0	294.87 ± 2.81	0.95	0.56
300	50:50	50:50	0.0	303.21 ± 2.94	0.96	0.57
		48:52	−2.0	307.81 ± 3.21	1.04	0.58

CV: coefficient of variance; R_f_: retardation factor.

## Sample Availability

Samples of the caffeine and paracetamol compounds are available from the authors.

## References

[1] Jimenez J.A., Martinez F. (2006). Thermodynamic study of the solubility of acetaminophen in propylene glycol + water cosolvent mixtures. J. Braz. Chem. Soc..

[2] Shakeel F., Alanazi F.K., Alsarra I.A., Haq N. (2012). Solubilization behavior of paracetamol in Transcutol-water mixtures at *T* = (298.15 to 333.15) K. J. Chem. Eng. Data.

[3] Shakeel F., Ramadan W. (2010). Transdermal delivery of anticancer drug caffeine from water-in-oil nanoemulsions. Colloids Surf. B.

[4] Rahimi M., Khorshidi N., Heydari R. (2020). Simultaneous determination of paracetamol and caffeine in aqueous samples by ultrasound-assisted emulsification microextraction coupled with high-performance liquid chromatography. Sep. Sci. Plus.

[5] Medina A.R., de Cordova M.L.F., Molina-Diaz A. (1999). Simultaneous determination of paracetamol, caffeine and acetylsalicylic acid by means of FI ultraviolet pls multioptosensing device. J. Pharm. Biomed. Anal..

[6] Tavallali H., Salami M. (2009). Simultaneous determination of caffeine and paracetamol by zero-crossing second derivative spectrophotometry in pharmaceutical preparations. Asian J. Chem..

[7] Tavallali H., Sheikhaei M. (2009). Simultaneous kinetic determination of paracetamol and caffeine by H-point standard addition method. Afr. J. Pure Appl. Chem..

[8] Aktas A.H., Kitis F. (2014). Spectrophotometric simultaneous determination of caffeine and paracetamol in commercial pharmaceutical by principal component regression, least squares and artificial neural networks chemometric methods. Croat. Chem. Acta.

[9] Uddin M.N., Mondol A., Karim M.M., Jahan R.A., Rana A.A. (2019). Chemometrics assisted spectrophotometric method for simultaneous determination of paracetamol and caffeine in pharmaceutical formulations. Bangladesh J. Sci. Ind. Res..

[10] Sebaiy M.M., Mattar A.A. (2020). H-point assay method for simultaneous determination of paracetamol and caffeine in panadol extra dosage forms. Can. J. Biomed. Res. Technol..

[11] Altun M.L. (2002). HPLC method for the analysis of paracetamol, caffeine and dipyrone. Turk. J. Chem..

[12] Issa I.M., Hassouna E.M., Zayed A.G. (2012). Simultaneous determination of paracetamol, caffeine, domperidone, ergotamine tartrate, propyphenazole, and drotaverine HCl by high performance liquid chromatography. J. Liq. Chromatogr. Rel. Technol..

[13] Tsvetkova B., Kostova B., Pencheva I., Zlatkov A., Rachew D., Peikov P. (2012). Validated method for simultaneous analysis of paracetamol and caffeine in model tablet formulation. Int. J. Pharm. Pharm. Sci..

[14] Cunha R.R., Chaves S.C., Ribeiro M.M.A.C., Torres L.M.F.C., Munoz R.A.A., Santos W.T.P.D., Richter E.M. (2015). Simultaneous determination of caffeine, paracetamol, and ibuprofen in pharmaceutical formulations by high-performance liquid chromatography with UV detection and by capillary electrophoresis with conductivity detection. J. Sep. Sci..

[15] Acheampong A., Gyasi W.O., Darko G., Apau J., Addai-Arhin S. (2016). Validated RP-HPLC method for simultaneous determination and quantification of chlorphenaramine maleate, paracetamol and caffeine in tablet formulation. Springer Plus.

[16] Narayanan V.L., Austin A. (2016). Determination of acetaminophen and caffeine using reverse phase liquid (RP-LC) chromatographic technique. Quest J. Res. Pharm. Sci..

[17] Aminu N., Chan S.-Y., Khan N.H., Farhan A.B., Umar M.N., Toh S.-M. (2019). A simple stability-indicating HPLC method for simultaneous analysis of paracetamol and caffeine and its application to determinations in fixed-dose combination tablet dosage form. Acta Chromatogr..

[18] Ali J.G., Muhammad I., Hamid S., Muhammad A.A., Shoaib H., Tasleem S. (2020). Simultaneous determination and quantification of paracetamol, caffeine and orphenadrine citrate using stability-indicating HPLC method in a fixed dose combination tablet dosage form. Ann. Pharmacol. Pharm..

[19] Belal F., Omar M.A., Derayea S., Zayed S., Hammad M.A., Saleh S.F. (2015). Simultaneous determination of paracetamol, caffeine and codeine in tablets and human plasma by micellar liquid chromatography. Eur. J. Chem..

[20] Wang A., Sun J., Feng H., Gao S., He Z. (2008). Simultaneous determination of paracetamol and caffeine in human plasma by LC-ESI-MS. Chromatographia.

[21] Tavallali H., Zareiyan S.F., Naghian M. (2011). An efficient and simultaneous analysis of caffeine and paracetamol in pharmaceutical formulations using TLC with a fluorescence plate reader. J. AOAC Int..

[22] Chabukswar A.R., Thakur V.G., Dharam D.L., Shah M.H., Kuchekar B.S., Sharma S.N. (2012). Development and validation of HPTLC method for simultaneous estimation of paracetamol, ibuprofen and caffeine in bulk and pharmaceutical dosage form. Res. J. Pharm. Technol..

[23] Halka-Grysinska A., Slazak P., Zareba G., Markowski W., Klimek-Turek A., Dzido T.H. (2012). Simultaneous determination of acetaminophen, propyphenazone and caffeine in cefalgin preparation by pressurized planar electrochromatography and high-performance thin-layer chromatography. Anal. Methods.

[24] Lau O.-H., Luk S.-F., Cheung Y.-M. (1989). Simultaneous determination of ascorbic acid, caffeine and paracetamol in drug formulations by differential-pulse voltammetry using a glassy carbon electrode. Analyst.

[25] Yigit A., Yardrm Y., Senturk Z. (2016). Voltametric sensor based on boron-doped diamond electrode for simultaneous determination of paracetamol, caffeine and aspirin in pharmaceutical formulations. IEEE Sens..

[26] Minh T.T., Phong N.H., Duc H.V., Khieu D.Q. (2018). Microwave synthesis and voltametric simultaneous determination of paracetamol and caffeine using an MOF-199-based electrode. J. Mater. Sci..

[27] Hung N.X., Quang D.A., Toan T.T.T., Dung N.N. (2020). The simultaneous determination of ascorbic acid, paracetamol, and caffeine by voltammetry method using cobalt Schiff base complex/SBA-15 modified electrode. ECS J. Solid State Sci. Technol..

[28] Ibrahim H., Hamdy A.M., Merey H.A., Saad A.S. (2021). Simultaneous determination of paracetamol, propyphenazone and caffeine in presence of paracetamol impurities using dual-mode gradient HPLC and TLC densitometry methods. J. Chromatogr. Sci..

[29] Meter M.I.V., Khan S.M., Taulbee-Cotton B.V., Dimmitt N.H., Hubbard N.D., Green A.M., Webster G.K., McVey P.A. (2021). Diagnosis of agglomeration and crystallinity of active pharmaceutical ingredients in over the counter headache medication by electrospray desorption ionization mass spectrometry imaging. Molecules.

[30] Katseli V., Economou A., Kokkinos C. (2020). A novel all-3D-printed cell-on-a-chip device as a useful electroanalytical tool: Application to the simultaneous voltametric determination of caffeine and paracetamol. Talanta.

[31] Boltia S.A., Soudi A.T., Elzanfaly E.S., Zaazaa H.E. (2020). Effect of genetic algorithm-based wavelength selection as a preprocessing tool on multivariate simultaneous determination of paracetamol, orphenadrine citrate, and caffeine in the presence of p-aminophenol impurity. J. AOAC Int..

[32] Muntean D.M., Alecu C., Tomuta I. (2017). Simultaneous quantification of paracetamol and caffeine in powder blends for tableting by NIR-chemometry. J. Spectrosc..

[33] Ortega-Barrales P., Padilla-Weigand R., Molina-Diaz A. (2002). Simultaneous determination of paracetamol and caffeine by flow injection-solid phase spectrometry using C_18_ silica gel as a sensing support. Anal. Sci..

[34] Kulikov A.U., Verushkin A.G. (2008). Simultaneous determination of paracetamol, caffeine, guaifenesin and preservatives in syrups by micellar LC. Chromatographia.

[35] Emre D., Ozaltrn N. (2007). simultaneous determination of paracetamol, caffeine and propyphenazone in ternary mixtures by micellar electrokinetic capillary chromatography. J. Chromatogr. B.

[36] Abdelrahman M.M., Abdelwahab N.S., Hegazy M.A., Fares M.Y., El-Sayed G.M. (2020). Determination of the abused intravenously administered madness drops (tropicamide) by liquid chromatography in rat plasma; an application to pharmacokinetic study and greenness profile assessment. Microchem. J..

[37] Duan X., Liu X., Dong Y., Yang J., Zhang J., He S., Yang F., Wang Z., Dong Y. (2020). A green HPLC method for determination of nine sulfonamides in milk and beef, and its greenness assessment with analytical eco-scale and greenness profile. J. AOAC Int..

[38] Pena-Pereira F., Wojnowski W., Tobiszewski M. (2020). AGREE-Analytical GREEnness metric approach and software. Anal. Chem..

[39] Foudah A.I., Shakeel F., Alqarni M.H., Alam P. (2021). A rapid and sensitive stability-indicating green RP-HPTLC method for the quantitation of flibanserin compared to green NP-HPTLC method: Validation studies and greenness assessment. Microchem J..

[40] Alam P., Salem-Bekhit M.M., Al-Joufi F.A., Alqarni M.H., Shakeel F. (2021). Quantitative analysis of cabozantinib in pharmaceutical dosage forms using green RP-HPTLC and green NP-HPTLC methods: A comparative evaluation. Sus. Chem. Pharm..

[41] (2005). International Conference on Harmonization (ICH), Q2 (R1): Validation of Analytical Procedures–Text and Methodology, Geneva, Switzerland.

